# Feeding young infants with their head in upright position reduces respiratory and ear morbidity

**DOI:** 10.1038/s41598-018-24636-0

**Published:** 2018-04-26

**Authors:** Avraham Avital, Milka Donchin, Chaim Springer, Shlomo Cohen, Efrat Danino

**Affiliations:** 1 Institute of Pulmonology, Hadassah University Hospital and Hadassah-Hebrew University Medical School, Jerusalem, Israel; 2 School of Public Health and Community Medicine, Hadassah University Hospital and Hadassah-Hebrew University Medical School, Jerusalem, Israel; 3 Head nurse, Pediatric Department Ein-Karem, Hadassah University Hospital and Hadassah-Hebrew University Medical School, Jerusalem, Israel

## Abstract

The influence of feeding position of the infant in the pathogenesis of ear and airway diseases has not been well established. We investigated the influence of instructing mothers to feed their 3-month old infants with their head in an upright position on ear and respiratory morbidity during a one-year follow-up. Mothers of 88 infants were instructed by trained nurses to feed their infants with their head in upright position (intervention group). The control group consisted of 75 mothers of infants of similar socioeconomic background who fed their infants without special instructions. Both groups were followed at Maternal-Child-Health clinics. Feeding position was evaluated at the beginning and the end of the twelve-month study, and parent reported morbidity data of both groups were evaluated at every 3-month’s follow-up meeting. Infants from the intervention group were fed at a more upright head position. Parameters of parent reported morbidity evaluated as area under the curve were significantly lower in infants from the intervention group concerning ear diseases, respiratory diseases, prolonged fever episodes, need of bronchodilator inhalations and antibiotic courses compared to the control group. Instructing mothers to feed infants with their head in upright position was accompanied with less morbidity and treatment burden.

## Introduction

Respiratory tract infections and otitis media are major causes of morbidity in infants and young children. Feeding infants and young children in supine body position has been connected with otitis media^1–4^ but no association has been reported between infant feeding position and respiratory or general morbidity.

Most infants are fed in a horizontal position during the first few months of life. During breast feeding, recommended positions for the mother are sitting and side-lying^5,6^, including the “cradle hold position” in which the infant is placed horizontally facing the mother’s breast and the “football hold” where the infant’s body is to the side of the mother. In these recommended positions, the infant is fed horizontally and most lactation consultants advise mothers to breastfeed their infants when they (mothers) lie down, as their main goal is to keep breastfeeding^7^. This tendency of feeding young infants in a horizontal position is also adopted for bottle-feeding. After the age of 5–7 months, infants start holding their bottle and might continue with bottle feeding at bed-time for 2–3 years.

Our hypothesis is that supine feeding position might be associated not only with ear problems but also with significant respiratory and general morbidity. We therefore investigated the effectiveness of instructing mothers to feed their infants in an upright head position since the age of three months and evaluated the morbidity of ear and respiratory tract during the following year.

## Methods

### Study Population

In Israel, preventative health care services are provided at Maternal-Child-Health (MCH) clinics network, with >95% compliance. These services include routine immunizations, feeding guidance, evaluation of growth and development and injury prevention programs. Children recruitment was done at two MCH clinics during 2011. All methods were carried out in accordance with relevant guidelines and regulations and all experimental protocols were approved by our local Ethic Committee (Hadassah University Center Helsinki Ethics Committee approval - 0349-10-HMO) and Clinical Trials registration (NCT03247946, August 14, 2017). Signed inform consent was obtained from one parent of each of the children recruited.

#### Sample size

Assuming a 20% change of feeding position in the intervention group and only 3% in the control group, a power criterion of 80% and an alpha error of 0.05 (two sided), the needed number of participants in the study was n = 124 (62 in each group). For an ongoing clinical research (1 year), we took a 20% molting effect and the final number of needed participants was n = 160 (80 in each group).

#### Inclusion criteria

All children bottle-fed at least once a day, visiting MCH clinics and parents agreeing to participate in the study.

#### Exclusion criteria

Prematurity, need of supplemental oxygen, any ear-throat-lung or airway congenital anomaly, congenital heart disease, any chronic disease of childhood or malignancy.

#### MCH clinics

The Health Ministry Regional Office randomly selected two clinics with similar geographic climate and socio-economic criteria. The clinics were allocated to intervention group (Rishon Le Zion) and control group (Rehovot).

### The intervention program included

All nurses of the intervention MCH clinic heard a detailed lecture presented by the author (A.A) explaining the hypothesis and aims of the study. The study coordinator, PhD student, (D.E) reinforced trained nurses and parents at each visit to the MCH clinic.

A poster (65 cm × 90 cm, Appendix 1) and a 12-minute video-film made by the author (A.A) explaining the hypothesis and recommendations were presented in the waiting room of the intervention MCH clinic. A copy of the video-film was given to each mother. Informed consent to publish the pictures of the children in the poster in an online open-access publication has been obtained from parents.

Reinforcement phone calls and SMS messages were sent by the author (D.E) to every mother during the 1-year follow-up.

### Control group program included

Nurses of the MCH clinic knew that their clinic served as a control group. Parents were not aware of the intervention program. Parents were explained that they will participate in a study concerning infant feeding and infant health. The same inclusion and exclusion criteria as in the intervention group were used.

### Data collection

A questionnaire was filled by the parent and included demographic, socioeconomic details, position of the infant during feedings and morbidity events reported by the parents that occurred during the month preceding every 3-month’s follow up visits to MCH clinic.

### Evaluation of intervention effectiveness

#### Feeding position

At the beginning and end of the study (first and last visit to MCH clinics), mothers pointed out the actual infant feeding position used for every meal. Four positions were shown to the parent: Supine (0° angle), head lying on pillow (30° angle), mid-position (45° angle) and upright position (90° angle).

#### Morbidity

1. Ear morbidity: Parent report of physician-diagnosed ear infection (otitis media) or ear fluids (serous otitis media) during the previous month.
2. Respiratory morbidity: Parent report of persistent cough, wheezing and physician-diagnosed bronchitis or pneumonia during the previous month.
3. Fever: Parent report of number of prolonged (at least 3-days) fever episodes during the previous month.
4. Antibiotics: Parent report of antibiotic courses taken during the previous month.
5. Inhalations: Parent report of bronchodilator (salbutamol, ipratropium) inhalations used during the previous month.

Morbidity and treatment variables were calculated as rate of occurrence during the preceding month of every 3-month’s meeting and as area under the curve (AUC) during the whole 1-year follow up for each variable.

#### Validation

Morbidity was validated in 10% of infants of both groups by the author (D.E) using medical records. Feeding position reports of the parents were validated by inspection of the author (D.E) at first and last meeting.

#### Statistics

Chi-square or exact Fisher tests were used for analysis of categorical variables. Means and standard deviation (SD) or standard errors (SE) were used for continuous variables. Linear regression analyses were used for predicting independent associates with morbidity (ear, respiratory and fever, as AUC). p < 0.05 was considered significant.

#### Data availability statement

The datasets generated during and/or analyzed during the current study are available from the corresponding author on reasonable request.

## Results

### Demographic data

The intervention group first included 90 children and 88 children ended the one-year follow-up. The control group first included 81 children and 75 children ended the one-year follow-up. Demographic data of the parents and the infants are presented in Table 1. There were no significant differences in the age of the parents, number of married couples and parent’s academic level (at least a bachelor’s degree) between the two groups. The mean number of children in the family was higher (p < 0.005) in the control group (2.3 ± 1.3) than in the intervention group (2.0 ± 0.8) with a male\female ratio of 1.08 and 0.85 respectively (p = 0.44).

**Table 1:** Demographic characteristics (mean ± SD) of the control and intervention groups.

	Control group (n = 75)	Intervention group (n = 88)	p value
Age of mother (yrs.)	31.1 ± 5.1	31.5 ± 4.2	0.70
Age of father (yrs.)	33.5 ± 5.2	34.0 ± 3.8	0.60
Married mother (%)	98.0	94.3	0.4
Mothers with academic education % (at least a bachelor’s degree)	89.3	79.6	0.17
Fathers with academic education % (at least a bachelor’s degree)	82.0	72.2	0.20
Number of children in family	2.3 ± 1.3	2.0 ± 0.8	0.005
Age of infant at recruitment (months)	3.2 ± 1.2	3.6 ± 1.0	0.04
Male\female ratio of children	1.08	0.85	0.44

### Type of feeding

There was no significant difference at the beginning or end of the study in the percentage of breast-fed infants or kind of formula used (p = 0.6 and 0.12, respectively), nor in the kind of feeding bottles or nipples between the groups (p = 0.22 and 0.16 respectively).

### Environmental factors

There was no significant difference between the two groups in the presence in day care center (p = 0.11), number of workers (p = 0.28) or number of infants (p = 0.11) in day care centers, number of bottle feedings per day (p = 0.64), amount of formula per meal at the beginning (p = 0.15) or end of the study (p = 0.48).

### Validation

There was a high correlation in pediatrician diagnoses (97%) between parent reports and checked medical records. There was a high correlation (92%) between parent reports of feeding position and author’s (D.E) observations.

### Feeding angle position

**Figure 1:**
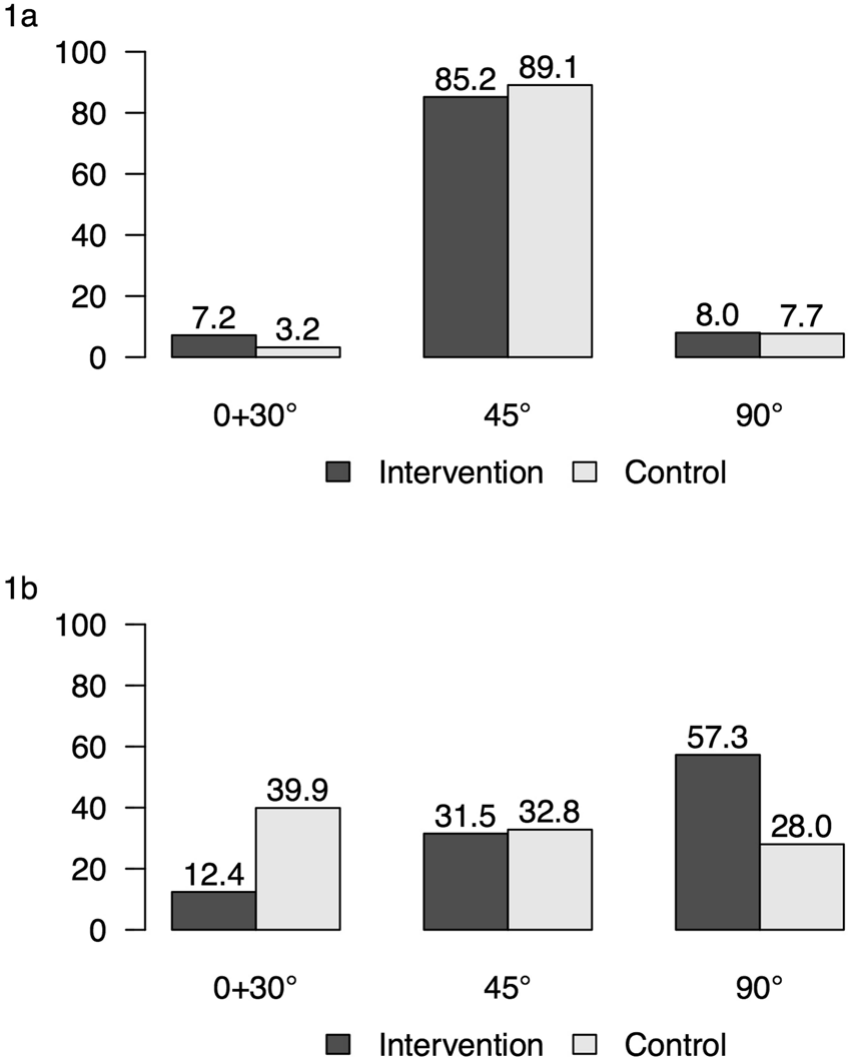
Percent of feeding positions of infants at beginning (**1a**) and end of study (**1b**) in intervention and control groups.

Positions supine (0°) and 30° were gathered to one group for statistical evaluation. At the beginning of the study (Fig. 1a), there was no significant difference between the rate of feeding position of both groups when the main position used was 45° in both groups (89.1% control group, 85.2% intervention group). At the end of the study (Fig. 1b), the most frequent position in the control group was 0°–30° (39.9% vs. 12.4% in the intervention group, p = 0.001) while in the intervention group, the most frequent position used was 90° (57.3% vs. 28.0% in the control group, p < 0.001). During the day, at the end of the study, the rate of children that were bottle-fed in at least 50% of their feedings at a 90° position was 62.5% in the intervention group and 30.7% in the control group (p < 0.001).

At the end of the study (children were 15 months-old), only a minority of children did eat during the night, 23 children in the control group and 19 children in the intervention group. 19 children from the control group ate during nights at position 0–30° compared to 9 children in the intervention group (p < 0.05).

### Morbidity

**Figure 2:**
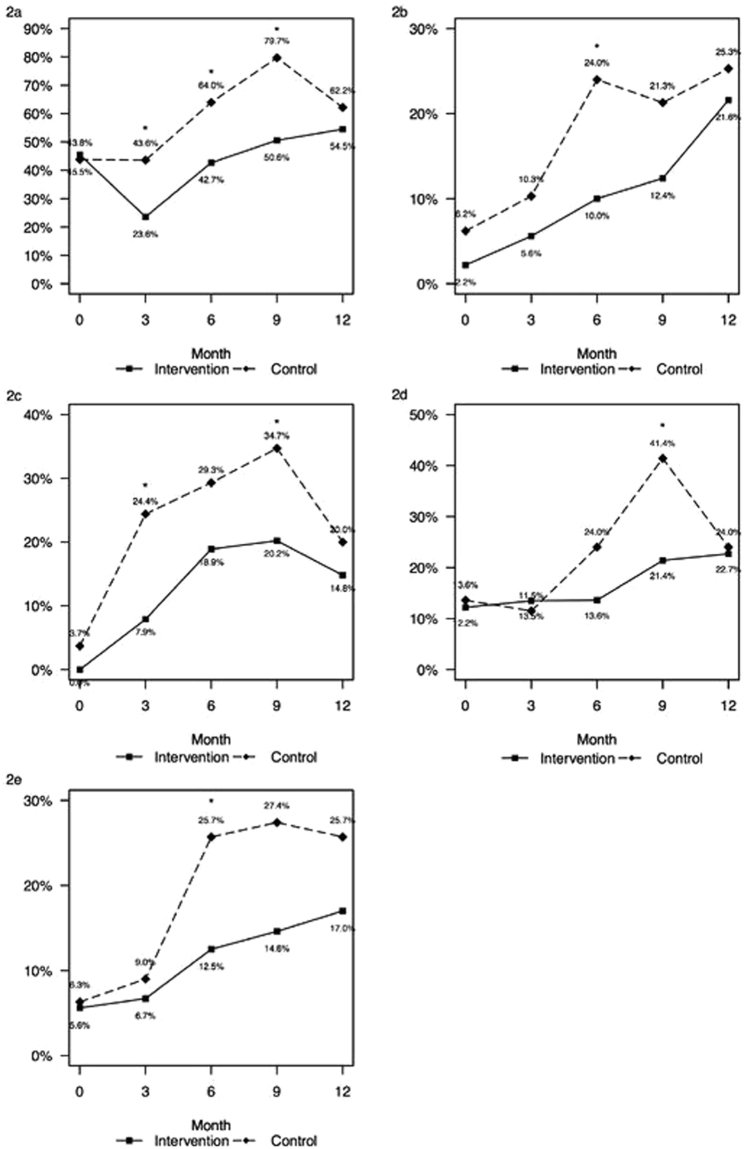
Rate of morbidity parameters reported by parents concerning the preceding month of every 3-month visit at MCH clinics**:** Respiratory morbidity (**2a**), ear morbidity (**2b**), prolonged fever episodes (**2c**), bronchodilator inhalation courses (**2d**) and antibiotic courses taken (**2e**). (asterisk for p < 0.05).

At the beginning of the study (mean age of the children was 3.4 months), there was no significant difference in the rate of occurrence of all parent reported morbidity parameters between the two groups. The rate of occurrence of respiratory morbidity (Fig. 2a) was significantly higher (asterisks) at months 3, 6 and 9 of follow up in the control group. The rate of occurrence of ear morbidity and of prolonged fever episodes in both groups are presented in Fig. 2b and 2c and the rate of bronchodilator (salbutamol, ipratropium) and antibiotic courses used in Fig. 2d and 2e. The rate of occurrence of the parameters evaluated was significantly higher in the control group in only part of the follow up periods, but the curves of all five parameters evaluated were higher in the control group. We therefore evaluated the area under the curve (AUC) of all morbidity parameters for every child during the 1-year follow-up and found that the mean AUC of all morbidity and treatment variables were significantly higher in the control group (Table 2).

**Table 2:** Area under the curve (AUC) of morbidity and treatment variables in intervention and control groups; means, standard error (SE) and statistical significance in control and intervention groups.

Morbidity parameters	Intervention	Control	p
	mean (SE)	mean (SE)	
Ears	0.50 (0.08)	0.81 (0.10)	<0.01
Respiratory	1.76 (0.13)	2.49 (0.12)	<0.001
Fever	0.63 (0.08)	1.07 (0.11)	<0.001
Inhalations	0.71 (0.10)	1.01 (0.12)	<0.05
Antibiotics	0.50 (0.08)	0.87 (0.11)	<0.005

Linear regression analyses (Table 3) for predicting independent associates with morbidity showed that the AUC for ear morbidity of the intervention group was lower by −0.218 (p = 0.011) than in the control group controlling for being in a day care. Removing the “group” (intervention\control) from the model (Model 2, Table 3) left the day care as the only predictor of ear morbidity. The AUC for respiratory morbidity and fever episodes was predicted significantly only by the “group”, showing a lower AUC for the intervention vs. the controls by −0.272 and −0.283 respectively, controlling for mother’s age, education, number of children, being in a day care and feeding position.

**Table 3:** Determinants of morbidity (Area under the curve) – linear regression.

Independent variables	Ear morbidity				Respiratory morbidity				Fever episodes			
	Model 1		Model 2		Model 1		Model 2		Model 1		Model 2	
	St.β	p value	St.β	p value	St.β	p value	St.β	p value	St.β	p value	St.β	p value
Mother’s age	−0.134	0.127	−0.164	0.065	0.173	0.052	0.135	0.135	−0.016	0.857	−0.055	0.544
Mother’s education^a^	−0.072	0.374	−0.076	0.356	0.003	0.974	0.977	0.977	−0.047	0.563	−0.052	0.533
No. of children	−0.029	0.738	0.021	0.805	−0.183	0.039	0.176	0.176	−0.102	0.248	−0.036	0.680
Child in a day care center^b^	0.210	0.009	0.216	0.009	−0.077	0.340	0.404	0.404	0.704	0.482	0.065	0.437
Feeding in 90^0^ in at least 50% of feedings^b^	0.032	0.705	−0.041	0.606	0.041	0.628	0.539	0.539	0.222	0.825	−0.076	0.355
Group (intervention vs. control)	−0.218	0.011			−0.272	0.002			−0.283	0.001		

St.β: Standardized Beta.

^a^Academic (at least a bachelor’s degree)/non-academic.

^b^At the end of the study.

## Discussion

In this manuscript, we have shown that instructing parents to feed their infants in an upright instead of a supine head position led to a significant mean change in the feeding position of the infants in the intervention group and to a significant decrease in parent reported morbidity of respiratory tract and middle ear infections, in the occurrence of prolonged fever episodes and in the need for bronchodilator inhalations and antibiotic courses.

Abnormal tympanography has been connected with supine feeding position^1–4^. Tully *et al*.^4^ performed tympanography before and after feeding a single bottle of formula in a supine or semi-supine position in ninety healthy children aged 7–24 months. Tympanography, that was normal, became abnormal in 59.6% and in 15% of the children after supine and semi-supine feeding position respectively. This change in tympanography after a single bottle of formula was attributed to formula entry into the middle ear. The increased incidence of formula entry to the middle ear in infants might be connected with a smaller angle of the eustachian tube^8^. In our study, we have found that infants from the control group had a significantly greater AUC of ear morbidity (Table 2).

The association between infant feeding position and respiratory morbidity has not been previously described. Mirrett^9^ demonstrated significant silent aspiration using swallow studies in most children (68.2%) with cerebral palsy. In order to prevent aspiration pneumonia in Intensive Care Units, Scolapio^10^ suggested elevating the head of the bed (45°), continuous subglottic suctioning and oral decontamination. In our study, we found a significant increase in the AUC of the respiratory morbidity (Table 2) in infants of the control group compared to the intervention group. Increased incidence of aspiration of liquid food in infants might be connected to the maturational descent of the epiglottis and larynx^11,12^. During the first few months of life, the larynx is high and close to the palate. Around the age of 4–6 months, the epiglottis and larynx descend from the palate, creating the oropharynx space which will help the newborn and child in the process of vocalization, but this space might also contribute to increased chances of aspiration of liquid food from the oropharynx. A more vertical head position during feeding in our intervention group may have contributed to a faster and safer passage of fluid food to the esophagus and to less chances of aspiration, with therefore less respiratory symptoms.

Another explanation to the increased tendency for respiratory problems in the control group is that supine feeding position might cause inflammation/infection in the posterior nasopharynx. Prolonged stasis of food, in the presence of local bacterial flora, might induce local lymph gland inflammation, especially of adenoids and tonsils. Both lymphatic glands are generally rudimentary in newborns, but in many infants, the adenoid tissue might grow silently during the first year of life. Prolonged stasis of food in the oro-nasopharynx seems to be the most plausible cause for the enlargement of these glands, especially of the adenoid gland. Tonsillar hypertrophy tends to appear later, most probably secondary to prolonged upper airway obstruction and infection. Upper airway obstruction by enlarged tonsils and adenoids has been associated with recurrent lower respiratory tract infections as has been shown by Konno^13^. In that study, the authors measured esophageal pressure during sleep studies in 19 children before and after adeno-tonsillectomy. They found, before surgery, elevation of the negative intra-thoracic inspiratory pressure to 4–6 times above normal values during deep sleep, returning to normal values after surgery. Contrast fluid (lipiodol) instilled into the oropharynx during sleep was aspirated into the airways and found in chest X-rays more frequently in children before (8/10 children) than after surgery (1/7 children). The authors concluded that active aspiration of secretions during deep sleep is due to upper airway obstruction. In our study, chronic supine feeding might have induced adenoid hypertrophy and increased inspiratory intra-thoracic negative pressure during sleep, recurrent aspiration of food, saliva and infected post-nasal drip. This might explain the increased incidence of parent reported episodes of cough, wheezing, bronchitis and pneumonia diagnoses, the increased occurrence of prolonged fever episodes and the increased use of bronchodilator inhalations and antibiotics in our control group.

In our study, children from the control group had more parent reported episodes of prolonged fever than children in the intervention group. This can be explained by more ear and respiratory tract infections. The children from the control group used also more bronchodilator inhalations than children from the intervention group. This is probably related to a higher frequency of respiratory symptoms in the control group. Children might sound wheezing or exhibit shortness of breath secondary to upper airway or bronchial secretions and may improve with bronchodilator inhalations. Children from the control group were also treated with more antibiotics courses than children from the intervention group.

It seems that in all the parameters we have used for the evaluation of parent reported infant morbidity, all were in the same direction: The control group exhibited a worse clinical picture, had more ear and respiratory infections, had more episodes of prolonged fever and needed more treatments with antibiotics and bronchodilator inhalations than the intervention group.

### Limitations of the study

The study was not blinded. There is a possibility that parents of the intervention group might have a tendency to report higher feeding position angles than the positions really used at home and might have a tendency to report lower rates of morbidity of their children, due to their enthusiasm with the trial.

There is also a possibility that some knowledge about the study in the intervention group might have reached parents from the control group since the two cities are only 15 km apart.

During the study, feeding position of the children was evaluated only at the beginning and at the end of the study (at the age of 3 and 15 months) and not at every 3-month follow up visit as was done with all morbidity parameters. This might be the reason why we could not find a direct correlation between feeding position angle and morbidity variables.

Further studies designed with adequate blinding are required to fully understand this issue.

In summary, instructing parents to feed young infants with their head in an upright instead of a horizontal position may be a simple and efficient way to protect them from substantial morbidity. The successful campaign against prone position of infants during sleep to prevent “Sudden Infant Death Syndrome” could be reinforced to “back to sleep and up to eat”.

“What is already known on this topic” – Supine feeding position has been associated with otitis media.

“What this study adds” - Feeding infants in upward head position was connected to less parent reported morbidity including: ear and respiratory infections, less episodes of prolonged fever and less use of antibiotic and bronchodilator inhalation courses.

## Electronic supplementary material

Appendix 
