# Supplementary material for: Feeding young infants with their head in upright position reduces respiratory and ear morbidity
Source: Sci Rep. 2018 Apr 26;8:6588. doi: 10.1038/s41598-018-24636-0 (PMC5920104; doi:10.1038/s41598-018-24636-0)
Supplement: Supplementary file 1 — Appendix [file 41598_2018_24636_MOESM1_ESM.pdf]

Appendix.1: Poster Up to eat, safe feeding.
Manuscript title: Feeding young infants with their head in upright position reduces respiratory and ear morbidity.
Authors: Avraham Avital, MD; Milka Donchin, MD; Chaim Springer, MD; Shlomo Cohen, MD and Efrat Danino, PhD.

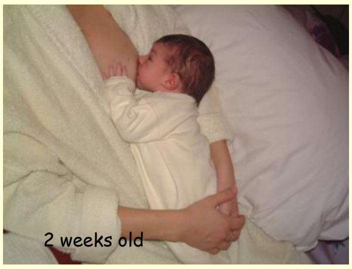
2 weeks old

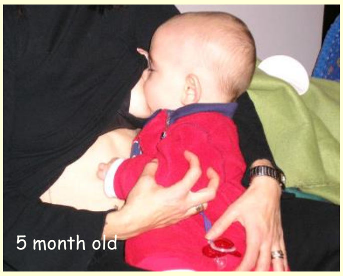
5 month old

Safe feeding

Prospective study
Hadassah University Hospital
Mother and Child Health Clinics
Helsinki Ethic Committee Agreement

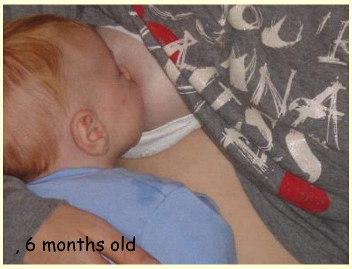
, 6 months old

Hypothesis:

Feeding in a supine position is associated with

1. Middle ear fluids and recurrent ear infections
2. Tonsil and adenoid hypertrophy, obstructive sleep apneas
3. Chronic cough, recurrent pneumonias, bouts of fever
4. Diffuse early dental caries

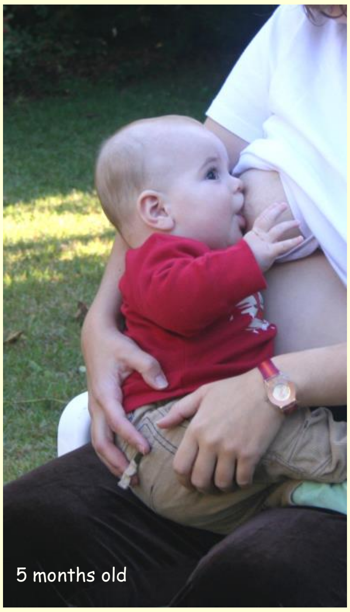
5 months old

Recommendations:

1. Get your child fed with his head in UPRIGHT position as soon as possible
2. Feed your child CAUTIOUSLY, without overfilling his mouth
3. Never feed your child when he is lying on his back
4. If the child is bottle-fed, it should be done by a PARENT, with the child's head always in an upright position
5. Switch as soon as possible to eat while SITTING around the table and drinking through an open cup.
6. Never get him used to take BOTTLE FEEDING at bed.
7. Keep ORAL HYGIENE as soon as possible before sleep.

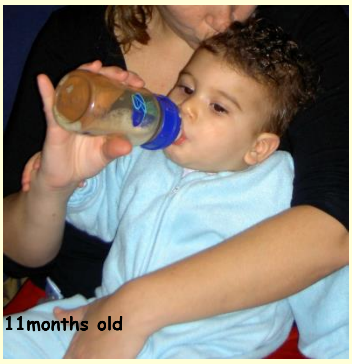
11months old

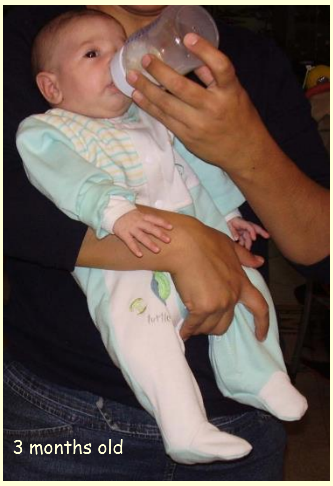
3 months old

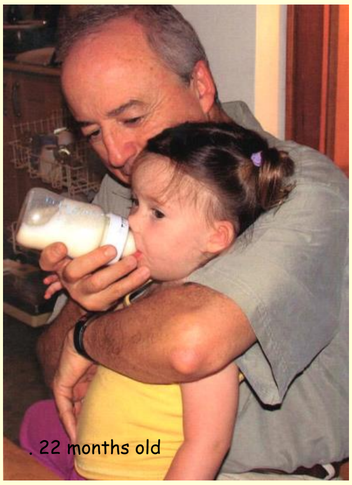
. 22 months old
